# Molecular generation by Fast Assembly of (Deep)SMILES fragments

**DOI:** 10.1186/s13321-021-00566-4

**Published:** 2021-11-14

**Authors:** Francois Berenger, Koji Tsuda

**Affiliations:** Graduate School of Frontier Sciences, The University of Tokyo, 5-1-5 Kashiwa-no-ha, Kashiwa, Chiba 277-8561 Japan

**Keywords:** Molecular generation, Molecular fragments, SMILES, DeepSMILES

## Abstract

**Background:**

In recent years, *in silico* molecular design is regaining interest. To generate on a computer molecules with optimized properties, scoring functions can be coupled with a molecular generator to design novel molecules with a desired property profile.

**Results:**

In this article, a simple method is described to generate only valid molecules at high frequency ($>300,000$ molecule/s using a single CPU core), given a molecular training set. The proposed method generates diverse SMILES (or DeepSMILES) encoded molecules while also showing some propensity at training set distribution matching. When working with DeepSMILES, the method reaches peak performance ($>340,000$ molecule/s) because it relies almost exclusively on string operations. The “Fast Assembly of SMILES Fragments” software is released as open-source at https://github.com/UnixJunkie/FASMIFRA. Experiments regarding speed, training set distribution matching, molecular diversity and benchmark against several other methods are also shown.

## Introduction

In recent years, there has been a surge of methods developed for in silico molecular generation. Mostly using deep neural networks [1–18], but not only [19–24]. Some authors use much simpler methods and the present contribution falls into this category. Notably, Polischuk [19, 25] uses molecular fragments and generates only valid molecules, while allowing some control [25] over the molecular diversity, novelty and synthetic complexity of the generated molecules. Kwon et al. [20] use direct cross-over and mutation operators over SMILES strings, combined with Conformational Space Annealing [26]. Their method does not require a training set but can generate invalid SMILES. Yoshikawa et al. [27] use a population-based grammatical evolution approach (ChemGE). While their method is fast and inherently parallel, it requires an initial population of molecules and can generate invalid SMILES. Nigam et al. [21] generate molecules by Gibbs sampling of SELFIES [28]. Their approach generates only valid molecules and does not require a training set. However, it requires translating molecules to/from SELFIES [28] (a recently developed linear encoding of molecular graphs). Brown et al. [23], Jensen et al. [22] and Leguy et al. [24] use a genetic algorithm over molecular graphs. Jensen’s method [22] doesn’t require task-specific model training and generates only valid molecules. Leguy et al. [24] use an evolutionary algorithm sequentially building a molecular graph using seven mutation operators. Their method also does not require a training set and generates only valid molecules.

The hereby proposed method works directly at the SMILES level. It generates only valid SMILES and thus valence-correct molecules. Simplified Molecular Input Line Entry System (SMILES [29]) is a molecular file format specifying a linear encoding of molecular graphs. SMILES are a compact way to store molecules on computers. The format is supported by all chemoinformatics toolkits and hence widespread. For rather small molecules, SMILES are human-readable. For the remaining of this article, it is only necessary to know that SMILES are strings made of balanced parentheses (indicating possibly nested branches of a linearized tree data structure), bracket atoms (an atom between brackets carries special properties^1^), digits indicating ring opening and closures on the molecular graph plus other characters listing atoms and the bonds between them. For more details, see the Open SMILES specification [30] or the seminal paper [29]. On the other hand, DeepSMILES [31] is a recently proposed variant of SMILES, designed to ease in-silico molecular generation by making it harder to generate syntactically invalid SMILES (also one of the goals of SELFIES [21]). DeepSMILES allows two options: (i) avoiding branch opening parentheses and/or (ii) avoiding ring opening numbers. In this study, only the DeepSMILES flavor without ring opening numbers is considered.

**Fig. 1:**
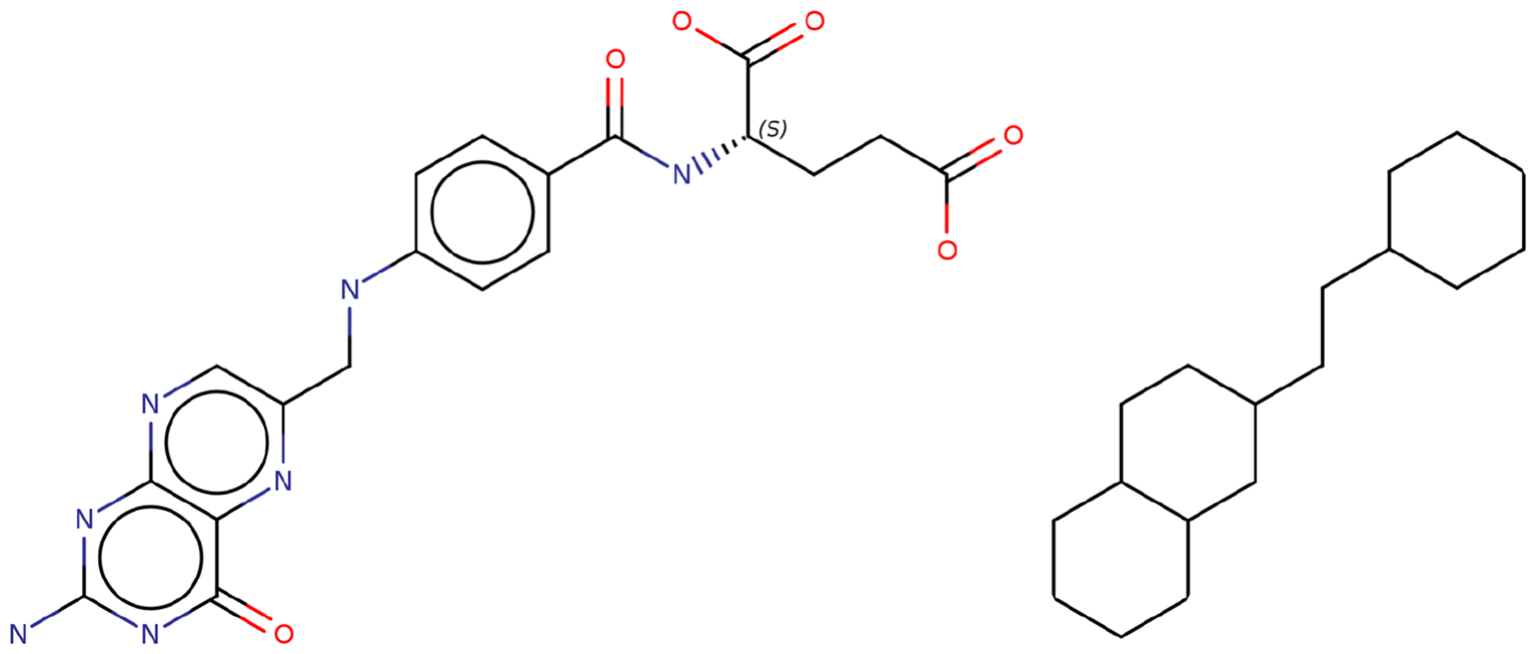
Folic acid (left) and its Bemis-Murcko scaffold (right)

In the experiments, to quantify molecular diversity in a dataset and the molecules generated from it, the count of unique Bemis-Murcko scaffolds [32] (Fig. 1) is monitored. In the remaining of this article, the datasets used, the method itself as well as computational experiments are presented and discussed.

## Methods

### The GuacaMol de Novo molecular design benchmark

GuacaMol [33–36] consists in a benchmark suite for molecular generators. The benchmark tasks measure the fidelity of models to reproduce the property distribution of a training set made of ChEMBL [37] 24 molecules^2^, the ability to generate novel molecules, the exploration and exploitation of the chemical space and several optimization tasks. In this study, since no molecular optimization was performed, only the molecular generation task was used. GuacaMol allows to compare performance against a few methods with published results, using a variety of metrics. All metrics are normalized; zero being the worst score and one the best.

GuacaMol metrics: *Validity* [33]: measures if the generated molecules are realistic (e.g. SMILES is valid according to RDKit [38]). *Uniqueness* [33]: assesses whether a model generates unique molecules (i.e. few or no duplicate canonical SMILES). *Novelty* [33]: assesses whether a model generates molecules which are not present in the training set. *Fréchet ChemNet Distance* [39]: measures how close the distributions of generated molecules are to training set ones. *Kullback-Leibler (KL) divergence* [40]: measures how well a probability distribution approximates another one. For this benchmark, the probability distributions of several physicochemical descriptors [33] are compared. This metric also captures molecular diversity (given a physicochemical property distribution, the generated molecules should be as diverse as training set ones).

Some methods with published GuacaMol results [33]: *Random sampler* [33]: a baseline model only random sampling the training set. *SMILES LSTM* [3, 33]: a Long-Short-Term Memory (LSTM) neural network that predicts the next character for partial SMILES strings. *Graph MCTS* [22, 33]: Jensen’s Graph-based Monte Carlo Tree Search molecular generator. *AAE*: an Adversarial AutoEncoder [41, 42]. *ORGAN*: Objective-Reinforced Generative Adversarial Network [41–43]. This deep-learning model architecture combines a generator and a discriminator network to generate molecules. *VAE*: a Variational AutoEncoder [41, 42]. This deep learning model learns a representation of molecules as latent vectors in a continuous space. The network architecture includes an encoder network that converts SMILES strings to latent vectors, and a decoder performing the reverse operation.

### Datasets

In the experiments, three datasets were used, in addition to the dataset internal to the GuacaMol benchmark. For the molecular generation speed benchmark, a random sample of one million molecules [44] from the GDB-13 [45] was used as the training set, so that results can be compared to the published results of Arús-Pous et al. [8]. For the molecular diversity and training set distribution matching experiments, two more datasets were used. To represent drug-like molecules, a bootstrap sample of 100,000 molecules was drawn from ChEMBL-28 [37]. To represent natural products, a bootstrap sample of 20,000 molecules was drawn from the Traditional Chinese Medicine Database at Taiwan [46] (this database is much smaller than ChEMBL).

The hereby proposed method is parameterized by a molecular fragmentation scheme and an atom typing scheme. Any molecular fragmentation scheme can be used, as long as it doesn’t cut rings (e.g. BRICS [47] or RECAP [48]). Many atom typing schemes could be used.

*Fragmenting molecules* In the experiments, the following ad-hoc fragmentation scheme was used to identify then select some cleavable bonds. Only single bonds between heavy atoms not in rings can be selected. Furthermore, the bond must not be connected to a stereo center nor involved in cis-trans isomerism (an attempt at preserving the stereochemistry of fragments, if present). Cleaved bonds are chosen randomly without replacement from this list, in order to obtain *n* fragments. By default, a fragment molecular weight (MW) of 150Da is used and the recommended number of fragments for molecule *m* is given by:

$$\begin{aligned} num\_frags(m) = round\left( \frac{MW(m)}{150}\right) \end{aligned}$$

This fragment weight parameter is just used as a hint to decide in how many fragments the given molecule must be cut (it is not strictly enforced). Since the fragmentation process is controlled by a random seed, if one requires more fragments from a given dataset, doing several passes with different seeds generates more fragments. As in Arús-Pous [9], randomized SMILES are used (instead of canonical ones) so that the same molecule does not always result in the same set of fragments (e.g. which fragment is a prefix of the SMILES, is the fragment written from left-to-right or the opposite). Duplicate fragments are not removed, because the fact that a fragment is found multiple times correlates with the natural occurring frequency of this fragment in a dataset. Also, removing duplicates might require a canonicalization step and would only be required (to reduce memory usage) if one is fragmenting a truly huge dataset.

*Typing atoms* In experiments and as in previous work [49], the following atom typing scheme inspired from atom pairs [50] was used: an atom $a_i$ in molecule *m* is identified by the tuple

$$\begin{aligned} type(a_i) = (\pi , e, h, f) \end{aligned}$$

where $\pi$ is the number of pi electrons, *e* the chemical element symbol, *h* the number of bonded heavy atoms and *f* the formal charge. Stereochemistry is ignored.

*Extended bond typing* While the bond order (BO) is a natural bond typing scheme, to better preserve some of the structure of a molecular training set^3^, it is useful to extend bond typing to make it more precise. Assuming $b_j$ is a bond in molecule *m* between atoms $a_i$ and $a_{i+1}$:

$$\begin{aligned} type(b_j) = (type(a_i), BO(b_j), type(a_{i+1})) \end{aligned}$$

Note that if $type(a_i) \ne type(a_{i+1})$, the bond is directed. In fact, several published methods use some kind of extended bond typing scheme (eMolFrag [51], CReM [19, 25]).

*Tagging cleaved bonds* Once some bonds have been selected for cleavage, this can be encoded into a valid SMILES string where unspecified atoms (the ‘*’ character in SMILES notation) are introduced and isotope numbers are used to encode the atom types of the atoms surrounding a cleaved bond. For example, tagging a cleaved bond in the SMILES for ethanol (’OCC’) would give: ‘O[2*][1*]CC’. Where isotope numbers one and two are indexes into an atom type dictionary (a mapping from integer to atom type). In the following, if a fragment is a prefix of a SMILES string, it is called a “seed fragment”. All other fragments are called “branch fragments”.

**Fig. 2:**
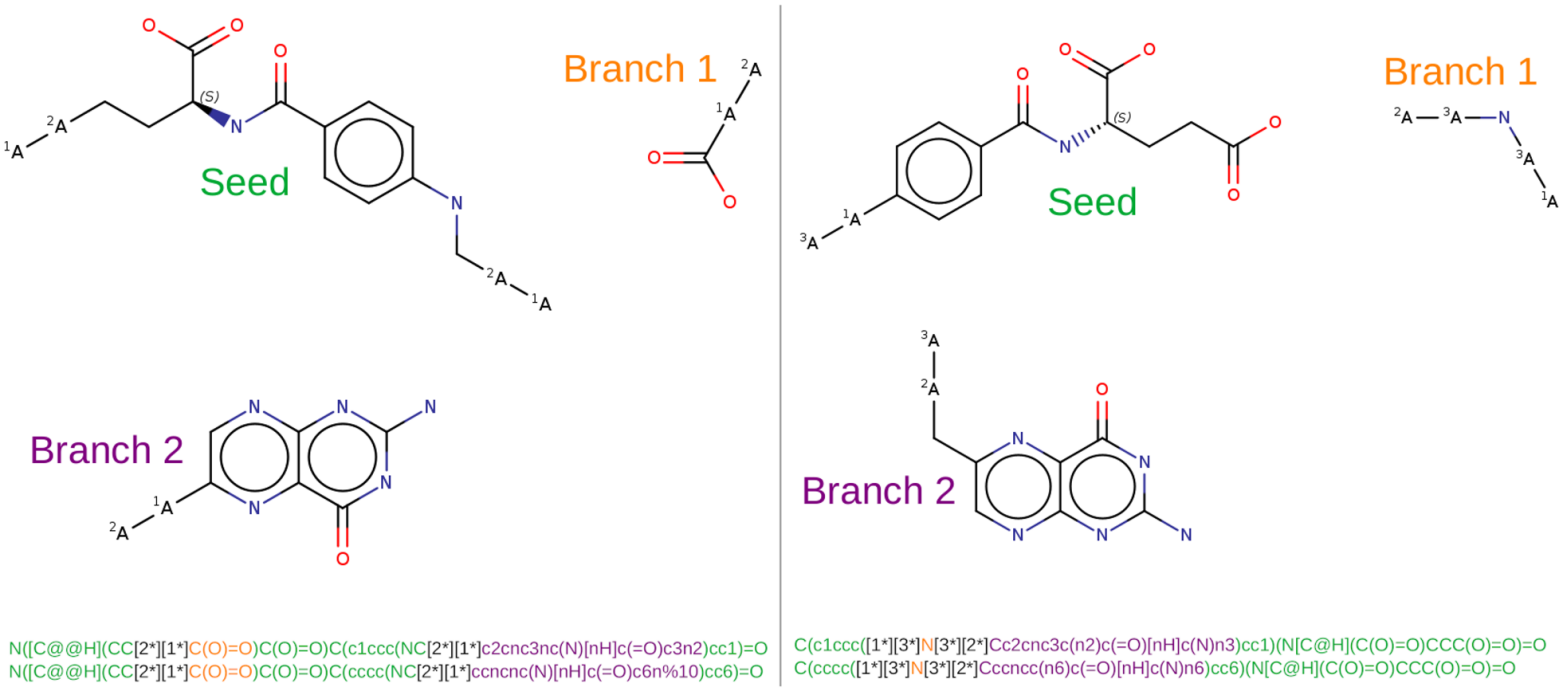
Two fragmentations of folic acid (vitamin B$_9$). Under each mixture of fragments, the (not canonical but randomized) SMILES and corresponding DeepSMILES-without-ring-openings are shown. Tagged cleaved bonds (in black) are the wildcard atom pairs [x*][y*] with the isotope numbers x and y encoding atom types directly to the left and right of the cleaved bond. Colors indicate the seed fragment (in green), the first branch fragment (in orange) and the second branch fragment (in purple)

**Fig. 3:**
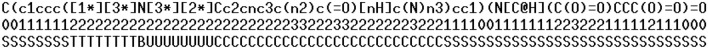
First line: SMILES string with tagged cleaved bonds. Second line: SMILES branch nesting depth of each character from the first line (0: not in a branch; 1: inside a branch; 2: inside a branch inside a branch, etc). Third line: S characters mark the “seed fragment”. T (resp. U) characters mark the first (resp. second) tagged cleaved bond. B marks the only heavy atom of the first “branch fragment”. C characters mark the atoms and bonds of the second “branch fragment”. The second “branch fragment” starts at SMILES branch nesting depth 2. This fragment cannot continue once the nesting depth becomes lower. The corresponding fragmented molecule can be seen on Fig. 2 right

*Fragmenting molecules with tagged cleaved bonds* A SMILES string with tagged cleaved bonds can be transformed into a mixture of fragments. This is not strictly necessary, as long as the molecular fragment assembler can recognize tagged cleaved bonds. However, this is useful to visualize the fragments with a 2D molecular viewer. The SMILES ‘O[2*][1*]CC’ becomes ‘O[2*][1*].[2*][1*]CC’. When extracting a fragment from a SMILES with tagged cleaved bonds, care must be taken to not extend the fragment past (or shorter than) its SMILES branch nesting depth. i.e. the nesting of parentheses pairs inside the SMILES string must be taken into account (Fig. 3).

*Indexing molecules with tagged cleaved bonds* For fast molecular generation, it is necessary to index all the fragmented molecules first. All seed fragments are stored into an array. Each branch fragment is stored into a hash-table of arrays where the prefix tagged cleaved bond is used as a key and the remaining of the fragment is appended to the array of all values for this key. For a fragment with several cleaved bonds, the first cleaved bond appearing in the fragment’s SMILES (when reading it from left to right) will determine under which cleaved bond type this fragment is registered. Our method uses randomized SMILES rather than canonical ones, in order to avoid any bias that could be introduced by the canonicalization procedure.

*Generating molecules* In essence, some kind of Markov sampling of the previously created data structure is performed, until the generated string has no tagged cleaved bond left. The algorithm is: (i) a seed fragment is uniformly drawn from the array of seed fragments. (ii) as long as the string under construction contains tagged cleaved bonds, each of them is deleted and replaced by a uniformly drawn fragment from the array of fragments with the tagged cleaved bond as the key. When generating the right flavor of DeepSMILES, such a fragment assembly algorithm can be performed using almost exclusively string operations. However, when generating SMILES, an extra step renumbering ring opening and closure numbers is required, to avoid number collisions between rings from different fragments.

*Software implementation* Typing atoms and bonds, selecting and tagging cleaved bonds is done by a Python script using RDKit [38]. For performance and correctness reasons [52], indexing fragments and fragment assembly is done by an OCaml [53] program reading SMILES with tagged cleaved bonds. The program is named FASMIFRA for “Fast Assembly of SMILES Fragments”.

## Results

**Fig. 4:**
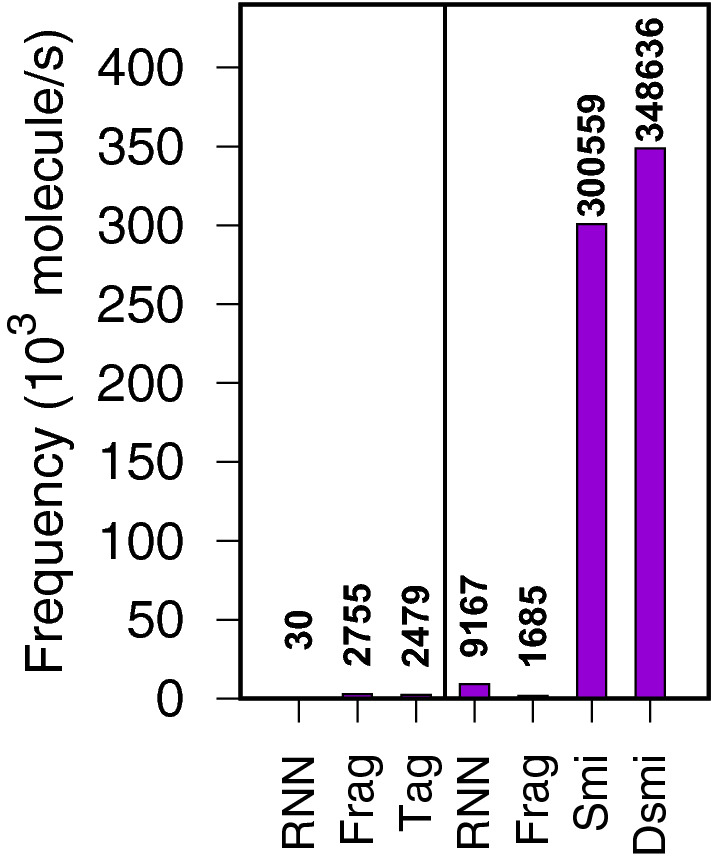
Model training (left) and sampling speed (right). RNN: Recurrent Neural Network (numbers cited from literature [8]); Frag (train): molecular fragmentation (Python/RDKit); Tag: tagging cleaved bond (proposed method, Python/RDKit); Frag (sampling): assembly of molecular fragments using molecular graph operations (Python/RDKit); Smi: fast assembly of SMILES fragments (proposed method, OCaml). Dsmi: fast assembly of DeepSMILES fragments (proposed method, OCaml); Tag is the model training prerequisite of Smi and Dsmi sampling. All methods use a single CPU, except RNN which uses four CPUs and one GPU

To assess the model training speed and molecular generation frequency of the proposed method, the 1M GDB-13 molecules sample [44] from Arús-Pous [8] was used and 2B molecules were generated (same protocol). The training set was fragmented (with a fragment molecular weight decreased from 150 to 50Da, because GDB-13 molecules are quite small) , then molecules were generated from those fragments (Fig. 4). Tests were run using a single core of an Intel Core i7 CPU @ 2.7GHz, with 12 cores and 16GB or RAM, under Ubuntu Linux 20.04 LTS.

To assess diversity of the generated molecules, as well as training set distribution matching, a sample of 100k molecules from ChEMBL-28 and a sample of 20k molecules from TCM@Taiwan were used. After molecular fragmentation of each set, the same number of molecules was generated (100k and 20k). The number of unique Bemis-Murcko scaffolds in each training and generated set is reported, along with the number at the intersection of those sets (Table 1).

**Table 1:** Molecular diversity assessed via the number of unique Bemis-Murcko scaffolds at the intersection between datasets

	ChEMBL_train (100k)	ChEMBL_gene (100k)	TCM_train (20k)	TCM_gene (20k)
ChEMBL_train (100k)	25135	8466 (23957 new $\sim =$ 74%)	979	982
ChEMBL_gene (100k)	8466 (23957 new $\sim =$ 74%)	32423	802	891
TCM_train (20k)	979	802	6056	2713 (5726 new $\sim =$ 68%)
TCM_gene (20k)	982	891	2713 (5726 new $\sim =$ 68%)	8439

To assess if the method is capable of training set distribution matching, those training and generated sets were projected into an eight dimensional space, where dimensions are quite unrelated (molecular weight, calculated LogP, #aromatic rings, topological polar surface area, #rotatable bonds, synthetic accessibility score [54], hydrogen bond acceptors and hydrogen bond donors). Then, the overlap between the training set and the corresponding generated set histogram was quantified using the Jaccard index (equation (1)). Let *X* and *Y* be two histograms with the same number of bins (*n*).

1 $$\begin{aligned} J(X, Y) = \frac{\sum _{1}^{n}\min (X_i,Y_i)}{\sum _{1}^{n}\max (X_i,Y_i)} \end{aligned}$$

**Fig. 5:**
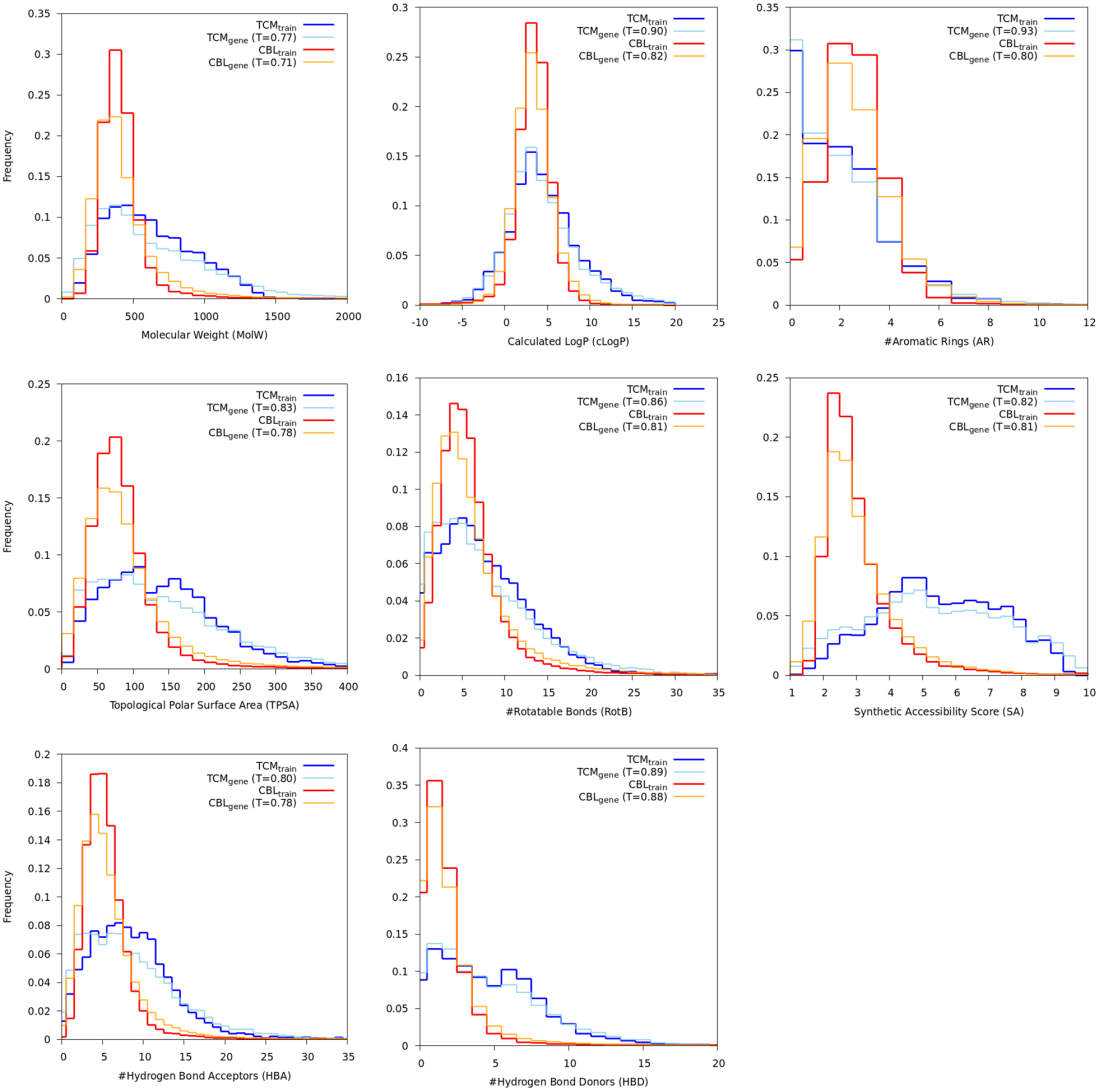
Distribution reproduction experiments’ histograms. Traditional Chinese Medicine database at Taiwan: training set in blue (TCM_train_), generated set in cyan (TCM_gene_). ChEMBL training set in red (CBL_train_), generated set in orange (CBL_gene_). The Jaccard index between the training and generated set histograms is shown in the legend (T = x)

A Jaccard index of zero means no overlap between two histograms, while one means perfect similarity (Fig. 5).

**Table 2:** Comparison of several molecular generators in the GuacaMol [33] distribution learning benchmark

Benchmark	Random sampler	SMILES LSTM	Graph MCTS	AAE	ORGAN	VAE	FASMIFRA	Negative control
Validity	1.000	0.959	1.000	0.822	0.379	0.870	1.000	1.000
Uniqueness	0.997	1.000	1.000	1.000	0.841	0.999	0.994	0.959
Novelty	0.000	0.912	0.994	0.998	0.687	0.974	0.702	0.947
KL_divergence	0.998	0.991	0.522	0.886	0.267	0.982	0.959	0.855
FCD	0.929	0.913	0.015	0.529	0.000	0.863	0.814	0.397

Random sampler: baseline model; SMILES LSTM: Long-Short-Term Memory DNN for SMILES strings; Graph MCTS: Graph-based Monte Carlo Tree Search; AAE: Adversarial AutoEncoder; ORGAN: Objective-Reinforced Generative Adversarial Network; VAE: Variational AutoEncoder; FASMIFRA: Fast Assembly of SMILES Fragments (proposed method); Negative control: FASMIFRA without extended bond typing (any fragment can be connected to any other fragment)

**Fig. 6:**
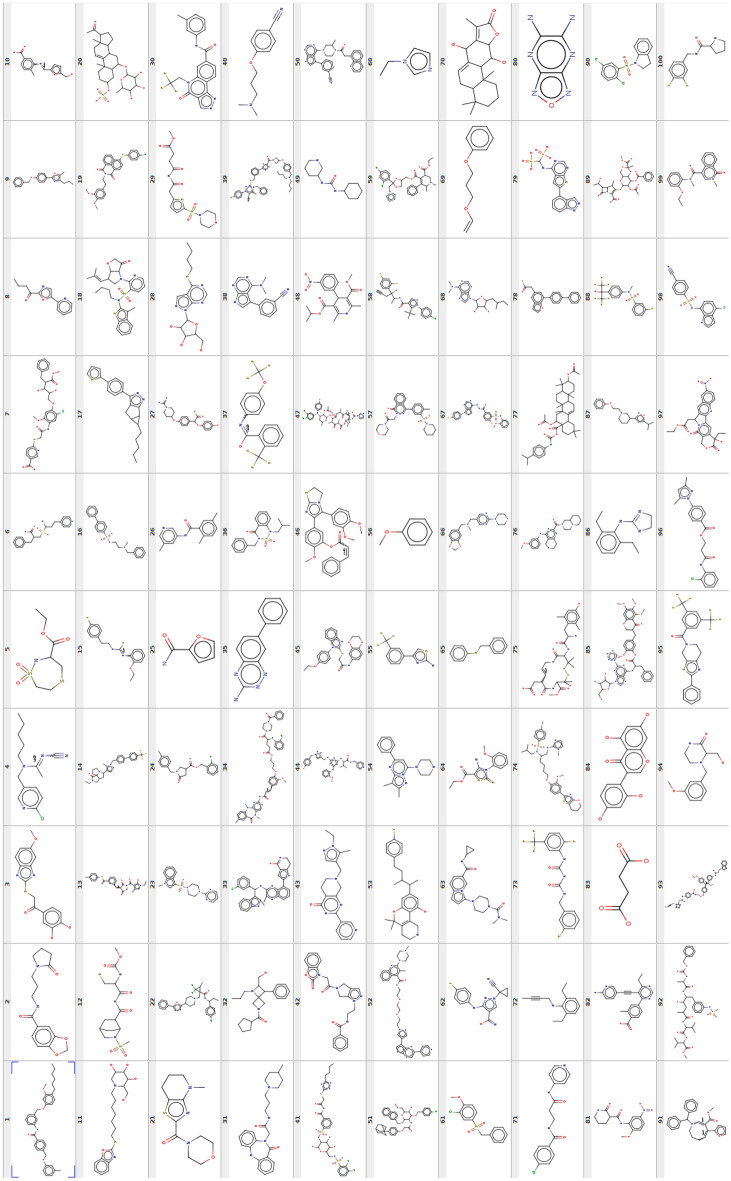
100 FASMIFRA-generated molecules during the GuacaMol benchmark (ChEMBL 24 training set)

Results on the GuacaMol molecular generation benchmark can be seen in Table 2 and Fig. 6.

## Discussion

*Model training frequency* (Fig. 4). RNN [8] is the slowest method, with $\sim$30 molecule/s (70 epochs; 8 min/epoch; 1M training set molecules). Plus, RNN used four CPUs and one GPU, so the per CPU processing frequency is much lower. Molecular fragmentation (2755 molecule/s) or cleaved bond tagging (proposed method; 2479 molecule/s); both written in Python using RDKit and running on a single CPU core have a more reasonable, and comparable, processing frequency.

*Model sampling frequency* (Fig. 4). Assembly of molecular fragments in Python using RDKit is the slowest method here ($\sim$1685 molecule/s). Editing a molecular graph (RWMol class in RDKit) is not so efficient. RNN is reasonably fast upon model sampling ($\sim$9167 molecule/s); although requiring four CPUs and one GPU. On the other hand, the proposed method of fast assembly of SMILES fragments reaches high sampling frequencies ($\sim$300599 SMILES-encoded molecule/s; $\sim$348636 for DeepSMILES).

*GuacaMol benchmark* (Table 2) In this benchmark, while FASMIFRA is one of the simplest methods (probably just after the Random sampler baseline model), a balanced performance profile is observed. As expected, FASMIFRA generates only valid molecules (Validity = 1.0). The only metric in which FASMIFRA is not great is Novelty (0.7); meaning that sometimes generated molecules are part of the training set. But, FASMIFRA being a fragment-based method, this was expected. Especially, extended bond typing constrains which fragment can be connected to which, effectively limiting the number of combinations which can be obtained from a fragment library. On the other hand, a negative control experiment was performed, where extended bond typing was turned off, which showed that while doing this improves the Novelty metric (from 0.702 to 0.947), this is at the detriment of training set distribution matching (KL_divergence is decreased from 0.959 to 0.855; FCD is decreased from 0.814 to 0.397). This negative control experiment shows that FASMIFRA is not a random molecular generator. Other methods which perform very well in the GuacaMol benchmark are the Random sampler baseline, but it cannot generate new molecules (Novelty = 0.0). The SMILES LSTM and the VAE are also very balanced and show good performance across all metrics. Compared to FASMIFRA, the Graph MCTS is lacking on the KL_divergence (0.522) and FCD metrics (0.015). The AAE is lacking on the FCD metric (0.529). The ORGAN is lacking on the Validity (0.379), KL_divergence (0.267) and FCD metrics (0.0). However, and to their defense, some of these methods might perform molecular optimization while FASMIFRA cannot (it would need to be coupled to a genetic algorithm or simulated annealer).

*Molecular diversity* (Table 1 and Novelty line in Table 2). In terms of Bemis-Murcko scaffolds, the proposed method generates a significant fraction of new scaffolds (74% in the generated set for ChEMBL; 68% in the generated set for TCM@Taiwan). With both datasets, generating molecules resulted in an increase of the number of unique Bemis-Murcko scaffolds in the generated set, compared to the training set (Table 1). As expected, the ChEMBL dataset (drug-like molecules) and the TCM@Taiwan dataset (natural products) are very different (Table 1 and Fig. 5). For example, both training sets share less than 1000 scaffolds.

*Training set distribution matching* (Fig. 5 and KL_divergence and FCD lines in Table 2). The method shows some propensity at training set distribution matching. For example, the minimum, median and maximum Jaccard indexes between histograms are (0.71, 0.805 and 0.88) for ChEMBL and (0.77, 0.845 and 0.93) for TCM@Taiwan.

On the positive side, this method is conceptually simple. Fragmenting molecules is reasonably fast (left of Fig. 4). Indexing fragments and generating molecules is extremely fast (right of Fig. 4). Only syntactically valid (Deep)SMILES encoded molecules are generated (Validity line in Table 2).

On the negative side, this method does require a training set. See the introduction for methods which don’t. Also, the method doesn’t create new rings. However, a medicinal chemistry technique is readily applicable in order to reasonably alter the generated rings [55]. If the training set contains molecules which cannot be fragmented (e.g. only made of fused rings), such molecules only contribute one seed fragment, without any attachable branch fragment (i.e. they might be copied as is from the training set to the generated set upon molecular generation). The method can generate duplicate molecules. Canonicalizing the produced SMILES would allow to detect and eventually filter those out.

## Conclusion

In this article, a simple method to generate molecules from molecular fragments was described. The method can work with any molecular fragmentation scheme, as long as rings are not opened/broken. Several experiments were presented, evaluating model training speed, molecular generation frequency, molecular diversity and training set distribution matching. The proposed method can be used as-is in genetic algorithm or simulated annealing fragment-based molecular generators. Our prototype software implementation is released under the GPL license. The technique may also be useful for dataset augmentation and demonstrates that DeepSMILES proposed useful simplifications to the SMILES syntax. We are not working on it and it might be difficult, but an interesting extension might be to support molecular fragmentation schemes which happen to open/break rings.

## Data Availability

The FASMIFRA software, TCM@Taiwan and ChEMBL training samples are on github: https://github.com/UnixJunkie/FASMIFRA (accessed 2021-07-28). The GDB-13 1M training sample can be downloaded from the GDB website [44, 45].

## Abbreviations

AAE: Adverserial AutoEncoder
DNN: Deep Neural Network
FASMIFRA: Fast Assembly of SMILES Fragments
GDB: Generated DataBase
LSTM: Long Short-Term Memory
MCTS: Monte Carlo Tree Search
ORGAN: Objective-Reinforced Generative Adversarial Network
RNN: Recurrent Neural Network
SELFIES: Self-referencing embedded strings [28]
SMILES: Simplified Molecular Input Line Entry System [29]
VAE: Variational AutoEncoder

## Footnotes

1 Like explicit hydrogens, a formal charge or a specific isotope number.

2 I.e. only molecules which have been synthesized and wet-lab tested.

3 Also, to prevent molecular generation from creating new/never-seen bonds and so to favor the synthesizability of the generated molecules.
